# Studying the Microanatomy of the Heart in Three Dimensions: A Practical Update

**DOI:** 10.3389/fped.2013.00026

**Published:** 2013-10-10

**Authors:** Jonathan C. Jarvis, Robert Stephenson

**Affiliations:** ^1^School of Sport and Exercise Sciences, Liverpool John Moores University, Liverpool, UK; ^2^Department of Musculoskeletal Biology, Institute of Ageing and Chronic Disease, University of Liverpool, Liverpool, UK

**Keywords:** micro-CT, cardiac anatomy, iodine contrast, 3-D visualisation, tractography, cardiomyocytes

## Abstract

The structure and function of the heart needs to be understood in three dimensions. We give a brief historical summary of the methods by which such an understanding has been sought, and some practical details of the relatively new technique of micro-CT with iodine contrast enhancement in samples from rat and rabbit. We discuss how the improved anatomical detail available in fixed cadaveric hearts will enhance our ability to model and to understand the integrated function of the cardiomyocytes, conducting tissues, and fibrous supporting structures that generate the pumping function of the heart.

## Introduction

The relationship between form and function in the heart has been studied by scientists for centuries. But the precise mechanism of the contractile function of the heart and its modes of failure continue to exercise the minds of scientists and clinicians in the present day. Close inspection of the heart by careful dissectors hinted at considerable complexity even before there was an understanding of the cellular basis of the structure, or of the fundamental mechanism of contraction. Pettigrew (1) introduced dissections that he presented to the Royal Society in 1859 as throwing light on the “Gordian knot” of anatomy, referring to a mythological challenge in the career of Alexander the Great. Alexander’s problem was that the ends of the rope that made up the knot were hidden. Pettigrew drew on the analogy: “the fibers of the ventricles, as a rule, have neither origin nor insertion; i.e., they are continuous alike at the apex of the ventricles and the base.” He observed the fibrous structure of the heart uncovering what he saw as seven identifiable layers by removing more superficial layers. He described his dissections with expert use of the English language, and with outstanding photography and line drawings, but he also recognized the importance of a three-dimensional understanding of the structures within the myocardium, and encouraged the reader to seek that understanding. He concluded his 1864 paper by describing a method to demonstrate his proposed alignment of the fibers by rolling up netting, or ruled paper and said “the author strongly recommends the use of these models, as a few minutes with such aids will throw more light on the course and direction of the fibers than hours of abstract reasoning without them.”

After 150 years the debate over the true microanatomy of the heart continues. We can now make virtual three-dimensional semi-transparent models of the internal structure of the ventricles based on the interaction of X-rays with that structure. Furthermore, we can obtain images not only from fixed and therefore lifeless material, but also from the beating heart with magnetic resonance imaging, computed tomography, and ultrasound scanning.

Physical dissection of the heart is informative, especially when guided by information from moving images and electrophysiological recordings from the beating heart. However, there is a level of structural information based on the disposition of individual cardiomyocytes, the genuine units of function, which is not accessible by simple dissection. For this level of understanding we must resolve at the scale of the single cell, at about 10 μm. The new method of micro-CT with iodine contrast enhancement also allows unprecedented discrimination between tissue types within the cardiac mass that is not possible by dissection.

We have worked toward new three-dimensional descriptions of cardiac anatomy based on micro-CT of cadaveric hearts for three main reasons:

## To Teach and to Facilitate Discussion Concerning Cardiac Anatomy

The normal structure and function of the heart, as well as the common pathological changes that cause abnormal function, consist in structures that operate in three dimensions. Therefore, interventions proposed to improve or restore cardiac function also require a three-dimensional appreciation of normal anatomy. Classical anatomical terminology may cause confusion in discussions or descriptions of particular anatomical dispositions, because the location of one structure is often described in terms of its complex relationship to other structures: for example, we might say “inferiolateral to the anterior cusp of the tricuspid.” While this terminology can be made unambiguous and universal across many spoken languages, it assumes detailed knowledge and is difficult to follow for non-specialists. Some of the sentences in Pettigrew’s (1) paper contain more than 100 words because he describes the course of fibers from base to apex, from endocardium to epicardium and from anterior to posterior. The success of numerical modeling projects requires groups of specialists to understand one another. Anatomists must address detailed anatomy with electrophysiologists, computer scientists, and mathematicians. The use of three-dimensional digital imaging improves such discussions; it removes the need to describe the physical structure in anatomical language in which the listener must rebuild the structure in his or her own mind. Rather, such discussions are greatly facilitated by access to three-dimensional views using software in which the virtual structure can be rotated, sliced, and manipulated to demonstrate one aspect or another of the substructure. Continuous structures can be labeled with a single color, angulation can be coded by false color mapping, and overlapping structures can be viewed by making overlying structures appear semi-transparent. Furthermore, when working with datasets that are fundamentally three-dimensional, such as computed tomographs, the information from layers above or below a point of interest often gives visual context that enables the human observer to detect patterns and boundaries that are very difficult to recognize in a series of two-dimensional slices. Furthermore, landmarks can be pinpointed within a few microns, and viewed in two-dimensional orthogonal planes and in a three-dimensional volume simultaneously.

## To Inform Numerical Models of Cardiac Function

There is a long history of simulation of the electrical activity of a heart by computing the transfer of depolarization between elements of a three-dimensional mesh or grid that represents the myocardium, and we cite just one representative example (2). Such a simulation requires an understanding of the excitability of the tissue elements themselves, and of the electrical properties of the material allowing conduction away from those elements in every direction. Such finite elements modeling of the heart has improved progressively as the anatomical information available from imaging techniques has improved. However, until now, no imaging technique except those based on analysis of two dimensional microscopic sections has been able to give an indication of structure at the level at which conduction is determined; that is, at the level of the individual cardiomyocytes and their inter-connections.

How the detailed prediction of electrical conduction is affected by the resolution of the finite elements mesh used for modeling remains to be determined. Many useful simulations of the distribution of electrical depolarization in the heart have been achieved with finite elements 10 or 20 times the size of individual heart cells (3). However, there is a good prospect now of successful cellular level models. These will incorporate information on the character of the action potential at each significant location, and predict directional propagation based not only on structure but also the known electrophysiological properties of the atrial myocytes, ventricular myocytes, nodal tissue, transitional tissue at the margins of the nodes, and specialized conducting tissue such as the purkinje fibers. As interest increases into the relationship between the fine structure of the heart muscle and the risk of arrhythmia, increasing fidelity of the underlying structure will be required. The increase in parallel computing structures within current commerce, research, and education suggests that numerical models that provide this level of detail will be manageable.

## To Guide Surgical Intervention and to Minimize Functional Deficit Post Surgery

Particularly in reconstructive surgery, pre-operative understanding of the cardiac anatomy makes surgery safer and quicker. Reconstruction of congenital abnormalities has been assisted greatly by the availability of pre-operative images produced by X-ray computed tomography. Cardiac ultrasound is also informative, and can now be achieved with catheter-based intra-cardiac probes. The position of the cardiac conduction system (CCS) is particularly important in reconstructive surgery, because key parts of the system run in areas of the cardiac structure that are also commonly involved in congenital defects (4, 5). Surgical damage to the atrio-ventricular part of the CCS, for example, may cause heart block requiring the use of a pacemaker, increasing both risk of complications and cost. Standard clinical imaging methods do not supply detail of the CCS, but new micro-CT methods based on fixed material with iodine contrast show great promise for three-dimensional analysis of this system in normal and malformed hearts (6). If three-dimensional images of whole hearts can be produced and disseminated with readily available viewing software, then these will act as interactive anatomical atlases for specific disease states that can be used to compare with images available for a particular patient, and to plan surgery to reduce risk.

### Three-dimensional microscopic anatomy in the heart

Individual cardiac myocytes are not long thin cylinders like skeletal muscle fibers. Observation of harvested myocytes from enzymatically digested myocardium shows that individual cardiomyocytes are rarely more than 20 μm in diameter, and about five times as long as they are wide. Streeter et al. (7) cut surfaces of fixed tissue, and studied ink prints made from those surfaces. But just as with the dissections of Pettigrew and others, the major visible structures are many times longer and wider than the individual cells. These structures, generally about five times the diameter of individual heart cells and bounded by connective tissue sheets are unhelpfully referred to in many published articles as fibers, or even myofibres. We agree with Lunkenheimer et al. (8) that it is important to recognize the basic unit of the contractile structure as the cardiomyocyte, and that larger structures presented to represent diffusion tensor MRI data in which the inherent isotropic resolution exceeds 25 μm, or to represent the results of physical dissections, are *aggregates* of cardiomyocytes.

### The move to three-dimensional descriptions of cardiac anatomy

To achieve a complete three-dimensional representation of the cardiac structure with microscopic resolution remains an enormous technical challenge. The studies of the Auckland group, using extended volume confocal microscopy and scanning electron microscopy are noteworthy. See, for example, (3, 9). While these results are impressive such techniques are destructive and are limited by small sample size. Gilbert et al. (10) have produced outstanding MRI images at 30 μm resolution, but this requires exposure times so long that deformation of the sample may be just as important a source of uncertainty as the inherent resolution of the scanner. Micro-CT offers a non-destructive, time efficient, and economical imaging modality for three-dimensional imaging of whole fixed hearts. Digital storage and graphics handling for high-resolution (and therefore very large) datasets has previously been the domain of specialist imaging instruments and imaging centers. However, the improvement in the handling of graphical information and numerical processing within the consumer market has now reached a level of development so that interactive data visualization is within the reach of every anatomical and physiological laboratory. Image presentation and analysis software is still a specialist niche, but we expect that viewer versions of the popular packages may become available, in an analogous way to the distribution of software to view, but not to edit, certain document formats. This will alleviate the problem that high-quality datasets are difficult to visualize in the standard format of contemporary scientific papers. The addition of video material can be helpful, but constrains the viewer to a particular aspect of the dataset. Just as the enormous datasets arising from genome wide analysis are being made freely accessible to the scientific community after publication, the same principle must surely soon apply to three-dimensional imaging datasets, so that interested parties can interrogate published data for themselves (with proper attribution in subsequent publications).

### Practical Considerations in Using Iodine Contrast for Micro Computed Tomography

We have found molecular iodine to be a practical and informative contrast agent in generating micro-CT images of biological soft tissue samples such as the heart. Our method is available in Stephenson et al. (6), but here we provide some additional practical hints and standard operating procedures to encourage more laboratories to make successful use of the technique.

**Figure 1:**
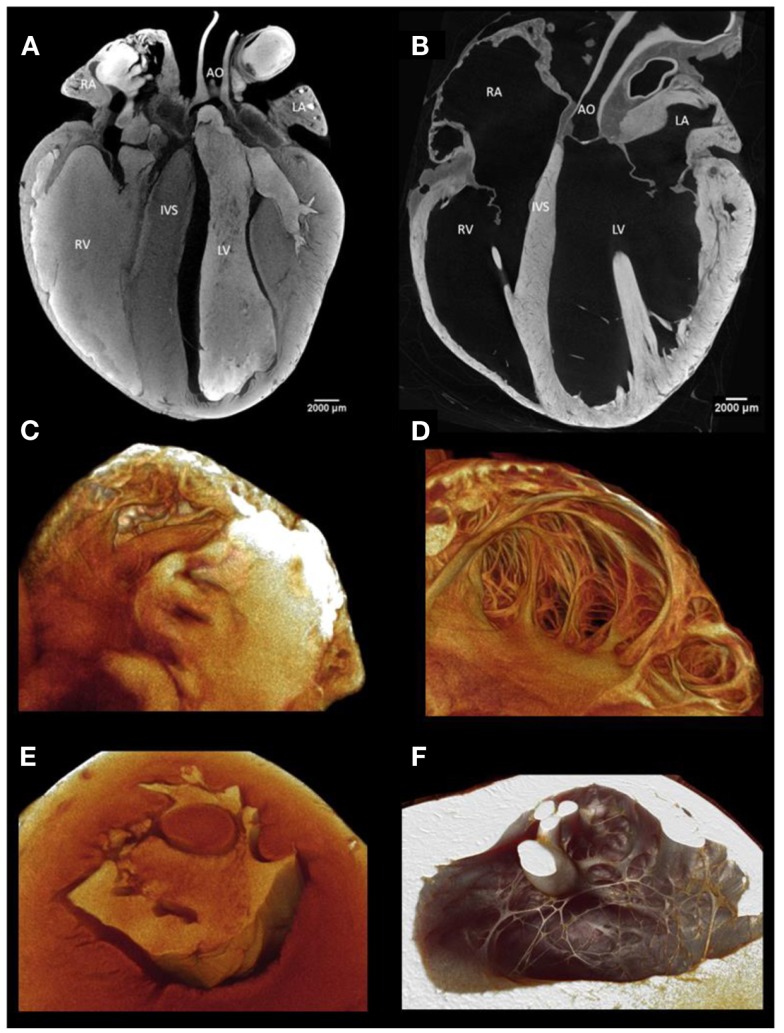
**(A)** A virtual longitudinal micro-CT section through a fixed rabbit heart stained with iodine contrast agent with blood residing in the cardiac chambers and great vessels. The residing blood shows a high attenuation because it has taken up iodine and thus the internal structure of the ventricular and atrial walls is not easily visualized. **(B)** This section illustrates the great improvement in anatomical discrimination achieved by removal of the blood by perfusion through the chambers and through the coronary circulation before fixation. Residing blood obscures visualization of the endocardial surface and cavity of atrial **(C)** and ventricular **(E)** samples. With careful flushing, perfusion fixation, and infusion with iodine, the endocardial surface and cavity of the atrium **(D)** and ventricle **(F)** are demonstrated in fine anatomical detail.

Image quality and access to the detailed morphology of internal structures of the heart is greatly improved by the removal of residing blood (Figure 1). Molecular iodine accumulates in clotted blood. Therefore, to achieve good delineation of blood-contacting surfaces, it is best to remove all blood from the cardiac chambers and blood vessels. We use systemic heparinization while blood is still circulating to extend the clotting time and to allow efficient washout of blood from tissues. After intravenous heparin, we allow circulation for several minutes and then perform euthanasia with an injectable anesthetic such as pentobarbitone (Euthatal, Merial Animal Health Ltd., Harlow, UK). The thoracic cavity is then opened by removing the anterior rib cage so that access to the heart is unimpeded. The aorta and inferior vena cava are cannulated with Teflon cannulae which are tied in place with linen sutures. In this configuration, the heart can be flushed with heparinized saline for several minutes via the vena cava and the aorta in turn, so that both the heart cavities and the coronary circulation are cleared of residing blood. When perfusing the aorta retrogradely, the surface of the heart is observed closely to confirm blanching of the cardiac tissue as clear fluid fills the coronary circulation. If blanching is not obvious this may be because the aortic valve is not closing sufficiently well to divert flow into the coronary arteries, and some constriction of the left ventricle may be necessary to encourage flow into the coronary circulation. It is helpful to tie off the superior vena cava while fluid is perfused into the inferior vena cava. This will prevent re-filling of the heart with blood from the upper body.

During perfusion, the heart should not be over-inflated above the size observed when the chest is opened immediately after death. For unimpeded examination of the internal surface of the heart, the next steps are designed to maintain an inflated state as well as possible during dissection, fixation, and infusion of the iodine contrast agent. This is particularly important for the atria which are challenging because of their lack of self-supporting structure.

We begin the fixation process by perfusing *in situ* with phosphate buffered formal saline via the same cannulae used to flush the sample. The tissue begins to gain some rigidity, although the atrial walls do not become sufficiently stiff to maintain their *in vivo* inflated state. Once fixative has been perfused for about 10 min, we therefore remove the heart with the great vessels attached and begin a further period of fixation *in vitro*, retaining the cannulae in the great vessels to inflate the chambers as necessary and using clips across the pulmonary artery and pulmonary veins to seal the chambers. The amount of filling, especially of the atria, is somewhat subjective – it would be difficult to try to maintain the *in vivo* situation at a state representing diastole by maintaining a constant pressure, for example, because fixation progressively stiffens the tissue and thus the pressure-volume characteristic is altered. In practice, as the tissue becomes fixed, it is usually sufficiently buoyant that the atria remain inflated as the whole heart floats in the fixation solution. Even in this situation, care must be taken to avoid loss of fluid from the chambers or flattening of the atria against the side of the containing vessel.

After fixation for a minimum of 24 h, the sample is placed in the appropriate iodine solution for a time that allows effective dispersal of the iodine within the tissue, but not so long that the tissue becomes saturated with iodine. Infusion with iodine is dependent on three main variables; sample size, iodine concentration, and time (11). Some guidelines for iodine administration into small mammalian hearts is available (6) but this aspect will inevitably require some trial and error.

Samples of human hearts for micro-anatomical study by this method are inevitably difficult to obtain. The most likely future sources are (a) from transplant operations for which the use of the explanted organ can be subject to an unambiguous consenting process and care can be taken to prepare the explants immediately after removal, or (b) from tissue that is donated for transplantation but in which some contra-indication for transplantation is identified, such as calcification of a valve. In some jurisdictions such material may properly be used for research or educational purposes. It is possible that the use of diffusible iodine may find application in virtual autopsy procedures. In this case, a period of perfusion with iodine solution followed by a period with clear perfusate to flush the vascular spaces might improve the resolution of cardiac imaging in post-mortem scans (12).

### Practical considerations for handling the datasets produced by micro-CT scanning

Our normal practice is to combine the use of Image J, an open access suite of programs, with the program Amira, (VSG, Merignac Cedex, France). One of the most important routine operations on such datasets is to “segment” the data, that is, to separate the anatomically relevant volume from the surrounding packaging (often thin sheets of polythene that may be required by local regulations for scanning biological samples in an imaging suite also used for non-biological samples) or to demarcate the biological tissue from enclosed space in scans of hollow organs like the heart. Segmentation may also be used to divide the scanned volume into biologically relevant sub-volumes such as the atria or the ventricles. Such sub-division is often necessary to allow numerical modeling to proceed by combining morphological information from tomography with functional properties from electrophysiological studies, immunohistochemical staining, or gene expression studies based on micro sampling (13–16). Furthermore, high-resolution Micro-CT produces data sets typically 10 GB per sample, and it may therefore be helpful to handle specimens in sub-volumes that are appropriate for the hardware available.

Segmentation of tomographic data is often described as laborious and not feasible for large N-numbers. This is true, especially with high-resolution data, if windowing is done slice by slice: spatial interpolation of a region of interest even over just a few slices will be inaccurate. Our approach to segmentation can save time. The terminology is based on the use of Amira, but the techniques are available in other commercial packages.

As an example, consider segmentation of the left ventricular free wall in a rabbit heart scanned in air at 18 μm resolution. If the free wall is approximately 20 mm from apex to base, that represents about 1100 slices. The myocardium is easily distinguished from the air space, but its surface is non-uniform. Therefore, if the myocardial area is outlined in one slice, interpolation of that boundary to the next 5 or 10 slices will result in errors of identification, because the interpolation algorithm is unable to accommodate the irregularity of the surface. Our method uses two stages: a primary segmentation based on inclusion of the wall with its immediately surrounding air volume, and a secondary segmentation based on differential attenuation.

The regions of interest (ROI) are initially selected using an oversized “brush” tool to incorporate both the ROI and a thin shell of empty space that just includes the outermost elements of the ROI. Using an large brush size that selects both ROI and surrounding regions of a significantly different set of pixel values (e.g., space) allows for interpolations between large numbers of slices (10–200), and makes for a highly efficient method. The selection is then stored, and the range of pixel values within the anatomically defined ROI are recorded. Using the “threshold” tool a masking window is then applied to the stored selection that identifies, and thus discriminates the ROI from the surrounding tissue or void space within the stored selection. The first selection is then removed leaving only the ROI pixels selected as the working volume.

### The relationship between micro-anatomical fidelity of structure and models of electrical depolarization

Many high fidelity models of the depolarization of the mammalian heart rely on the three-dimensional datasets available from magnetic resonance scans using the diffusion tensor imaging technique. Strictly speaking this is not a morphological method in which structures such as cell boundaries are imaged by differential attenuation. Rather, the preferential diffusion direction for water is calculated for each voxel, and since water is less constrained to move in the long axis than in the short axis of a fiber, then an indication of fiber direction is achieved. However, many papers are unclear about the resolution achieved or claimed with this class of techniques. The volumetric resolution of the technique in terms of the unit volume scanned is commonly about 400 μm. Some techniques can infer from the probability density function obtained from each voxel a proportion of that volume running in particular directions and this calculation has been represented as streamlines representing the distribution of direction of diffusibility. This is still not the same as visualizing the cellular structure, and a controversy is currently playing out concerning the proposed arrangement of nerve fibers in the brain based on similar analysis (17–19).The virtual fibrous structures achieved by this technique are usually of the order of 400 μm in diameter although Gilbert et al. (10) have published magnetic resonance imaging of a rat heart with 25 μm × 25 μm × 37 μm resolution using gadolinium uptake to generate contrast. To achieve such resolution, however, scan times of several days were required.

By contrast, micro-CT imaging is potentially capable of producing a fiber direction matrix at the resolution of the single myocyte based on contrast between the myocyte and the surrounding endomysium (6, 11). This distribution is inevitably more complex given its finer resolution than the equivalent DTI distribution and can potentially reflect changes in structure such as an increase in perimysial connective tissue, though scanning electron microscopy and extended volume confocal microscopy will continue to provide the greatest resolution for some time to come, but limited to very small samples. We (in collaboration with colleagues in Manchester UK and Auckland NZ) have recently begun to extract fiber direction maps from three-dimensional micro-CT scans by means of the structure tensor methods originally used to analyze the complex structure within sheets of paper or reconstituted wood products (20), and more recently developed to extract fiber orientation of cardiac tissue from data generated by episcopic microscopy (21, 22). In this technique, an algorithm samples the space surrounding an individual voxel and searches for the direction in which the attenuation changes the least. In this way each voxel of the original dataset is assigned a vector in three dimensions. By adjusting the parameters of the algorithm, these vectors may be used to build up a representation of the direction of individual cardiomyocytes as they pass through the voxels that make up the scanned volume. While this technique is operating at the limit of resolution presently afforded by micro-CT, it is based on attenuation data that derives directly from the physical structure of the biological material under scrutiny. Such rich data can thus be used to investigate the propagation of electrical activity within a close numerical representation of the real cardiomyocyte structure, including complex areas such as the trabecular endocardial surface of the mammalian heart. Electrophysiological data on the differential membrane properties that arise from non-uniform distribution of channel proteins with high-resolution maps of structure, will provide even better predictions of normal conduction and the vulnerability of abnormal structures or channel distributions to arrhythmias. Some illustrations of our first trials with this technique can be found in (22, 23).

## Conclusion

Micro-CT with iodine contrast requires careful sample preparation, but offers the best combination of anatomical detail and three-dimensional coverage presently available. We have offered some practical suggestions that we hope will assist other groups in generating high-quality images of biological samples.

## Conflict of Interest Statement

The authors declare that the research was conducted in the absence of any commercial or financial relationships that could be construed as a potential conflict of interest.
